# Supplementary material for: Comparing pregnancy and pregnancy outcome rates between adolescents with and without pre-existing mental disorders
Source: PLoS One. 2024 Mar 14;19(3):e0296425. doi: 10.1371/journal.pone.0296425 (PMC10939254; doi:10.1371/journal.pone.0296425)
Supplement: S1 Table — The list of ICD-9-CM codes to indicate prenatal visits, pregnancy outcomes, or delivery. (DOCX) [file pone.0296425.s001.docx]

| S1 Table. Pregnancy-related ICD-9-CM Codes | | | |
| --- | --- | --- | --- |
| Category | Code Type | Code | Description |
| Livebirth | CPT | 99440 | Newborn resuscitation: provision of positive pressure ventilation and/or chest compressions in the presence of acute inadequate ventilation and/or cardiac output |
|  | CPT | 99465 | Newborn resuscitation: provision of positive pressure ventilation and/or chest compressions in the presence of acute inadequate ventilation and/or cardiac output |
|  | ICD9CM | V270 | Mother with single liveborn |
|  | ICD9CM | V272 | Mother with twins both liveborn |
|  | ICD9CM | V273 | Mother with twins one liveborn and one stillborn |
|  | ICD9CM | V275 | Mother with other multiple birth all liveborn |
|  | ICD9CM | V276 | Mother with other multiple birth some liveborn |
|  | ICD9CM | V30 | Single liveborn |
|  | ICD9CM | V300 | Single liveborn, born in hospital |
|  | ICD9CM | V3000 | Single liveborn, born in hospital, delivered without mention of cesarean section |
|  | ICD9CM | V3001 | Single liveborn, born in hospital, delivered by cesarean section |
|  | ICD9CM | V301 | Single liveborn, born before admission to hospital |
|  | ICD9CM | V302 | Single liveborn, born outside hospital and not hospitalized |
|  | ICD9CM | V31 | Twin birth, mate liveborn |
|  | ICD9CM | V310 | Twin, mate liveborn, born in hospital |
|  | ICD9CM | V3100 | Twin birth, mate liveborn, born in hospital, delivered without mention of cesarean section |
|  | ICD9CM | V3101 | Twin birth, mate liveborn, born in hospital, delivered by cesarean section |
|  | ICD9CM | V311 | Twin birth, mate liveborn, born before admission to hospital |
|  | ICD9CM | V312 | Twin birth, mate liveborn, born outside hospital and not hospitalized |
|  | ICD9CM | V3201 | Twin birth, mate stillborn, born in hospital, delivered by cesarean section |
|  | ICD9CM | V321 | Twin birth, mate stillborn, born before admission to hospital |
|  | ICD9CM | V322 | Twin birth, mate stillborn, born outside hospital and not hospitalized |
|  | ICD9CM | V34 | Other multiple birth (three or more), mates all liveborn |
|  | ICD9CM | V340 | Other multiple, mates all liveborn, born in hospital |
|  | ICD9CM | V3400 | Other multiple birth (three or more), mates all liveborn, born in hospital, delivered without mention of cesarean section |
|  | ICD9CM | V3400 | Other multiple birth (three or more), mates all liveborn, born in hospital, delivered without mention of cesarean section |
|  | ICD9CM | V341 | Other multiple birth (three or more), mates all liveborn, born before admission to hospital |
|  | ICD9CM | V342 | Other multiple birth (three or more), mates all liveborn, born outside hospital and not hospitalized |
|  | ICD9CM | V36 | Other multiple birth (three or more), mates liveborn and stillborn |
|  | ICD9CM | V36 | Other multiple birth (three or more), mates liveborn and stillborn |
|  | ICD9CM | V360 | Other multiple, mates liveborn and stillborn, born in hospital |
|  | ICD9CM | V360 | Other multiple, mates liveborn and stillborn, born in hospital |
|  | ICD9CM | V3600 | Other multiple birth (three or more), mates liveborn and stillborn, born in hospital, delivered without mention of cesarean section |
|  | ICD9CM | V3600 | Other multiple birth (three or more), mates liveborn and stillborn, born in hospital, delivered without mention of cesarean section |
|  | ICD9CM | V3601 | Other multiple birth (three or more), mates liveborn and stillborn, born in hospital, delivered without mention of cesarean section |
|  | ICD9CM | V3601 | Other multiple birth (three or more), mates liveborn and stillborn, born in hospital, delivered without mention of cesarean section |
|  | ICD9CM | V361 | Other multiple birth (three or more), mates liveborn and stillborn, born before admission to hospital |
|  | ICD9CM | V37 | Other multiple birth (three or more), unspecified whether mates liveborn or stillborn |
|  | ICD9CM | V370 | Other multiple, unspecified, born in hospital |
|  | ICD9CM | V3700 | Other multiple birth (three or more), unspecified whether mates liveborn or stillborn, born in hospital, delivered without mention of cesarean section |
|  | ICD9CM | V3701 | Other multiple birth (three or more), unspecified whether mates liveborn or stillborn, born in hospital, delivered by cesarean section |
|  | ICD9CM | V371 | Other multiple birth (three or more), unspecified whether mates liveborn or stillborn, born before admission to hospital |
|  | ICD9CM | V372 | Other multiple birth (three or more), unspecified whether mates liveborn or stillborn, born outside of hospital |
|  | ICD9CM | V39 | Liveborn, unspecified whether single, twin, or multiple |
|  | ICD9CM | V390 | Other liveborn, unspecified, born in hospital |
|  | ICD9CM | V3900 | Liveborn, unspecified whether single, twin or multiple, born in hospital, delivered without mention of cesarean section |
|  | ICD9CM | V3901 | Liveborn, unspecified whether single, twin or multiple, born in hospital, delivered by cesarean section |
|  | ICD9CM | V391 | Liveborn, unspecified whether single, twin or multiple, born before admission to hospital |
|  | ICD9CM | V392 | Liveborn, unspecified whether single, twin or multiple, born outside hospital and not hospitalized |
| Stillbirth | CPT | 88016 | Necropsy (autopsy), gross examination only; macerated stillborn |
|  | HCPCS | S2265 | Induced abortion, 25 to 28 weeks |
|  | HCPCS | S2266 | Induced abortion, 29 to 31 weeks |
|  | HCPCS | S2267 | Induced abortion, 32 weeks or greater |
|  | ICD9CM | V271 | Mother with single stillborn |
|  | ICD9CM | V274 | Mother with twins both stillborn |
|  | ICD9CM | V277 | Mother with other multiple birth all stillborn |
|  | ICD9CM | 65641 | Intrauterine death affecting management of mother delivered |
|  | ICD9CM | V32 | Twin birth, mate stillborn |
|  | ICD9CM | V320 | Twin, mate stillborn, born in hospital |
|  | ICD9CM | V3200 | Twin birth, mate stillborn, born in hospital, delivered without mention of cesarean section |
|  | ICD9CM | V35 | Other multiple birth (three or more), mates all stillborn |
|  | ICD9CM | V350 | Other multiple, mates all stillborn, born in hospital |
|  | ICD9CM | V3500 | Other multiple birth (three or more), mates all still born, born in hospital, delivered without mention of cesarean section |
|  | ICD9CM | V3501 | Other multiple birth (three or more), mates all still born, born in hospital, delivered by cesarean section |
|  | ICD9CM | V351 | Other multiple birth (three or more), mates all stillborn, born before admission to hospital |
|  | ICD9CM | V352 | Other multiple birth (three or more), mates all stillborn, born outside of hospital and not hospitalized |
|  | ICD9CM | V36 | Other multiple birth (three or more), mates liveborn and stillborn |
|  | ICD9CM | V360 | Other multiple, mates liveborn and stillborn, born in hospital |
|  | ICD9CM | V3600 | Other multiple birth (three or more), mates liveborn and stillborn, born in hospital, delivered without mention of cesarean section |
|  | ICD9CM | V3601 | Other multiple birth (three or more), mates liveborn and stillborn, born in hospital, delivered without mention of cesarean section |
|  | ICD9CM | V362 | Other multiple birth (three or more), mates liveborn and stillborn, born outside hospital and not hospitalized |
| Abortion | CPT | 1966 | Anesthesia for induced abortion procedures |
|  | CPT | 59840 | Induced abortion, by dilation and curettage |
|  | CPT | 59841 | Induced abortion, by dilation and evacuation |
|  | CPT | 59850 | Induced abortion, by 1 or more intra-amniotic injections (amniocentesis-injections), including hospital admission and visits, delivery of fetus and secundines |
|  | CPT | 59851 | Induced abortion, by 1 or more intra-amniotic injections (amniocentesis-injections), including hospital admission and visits, delivery of fetus and secundines; with dilation and curettage and/or evacuation |
|  | CPT | 59852 | Induced abortion, by 1 or more intra-amniotic injections (amniocentesis-injections), including hospital admission and visits, delivery of fetus and secundines; with hysterotomy (failed intra-amniotic injection) |
|  | CPT | 59855 | Induced abortion, by 1 or more vaginal suppositories (eg, prostaglandin) with or without cervical dilation (eg, laminaria), including hospital admission and visits, delivery of fetus and secundines |
|  | CPT | 59856 | Induced abortion, by 1 or more vaginal suppositories (eg, prostaglandin) with or without cervical dilation (eg, laminaria), including hospital admission and visits, delivery of fetus and secundines; with dilation and curettage and/or evacuation |
|  | CPT | 59857 | Induced abortion, by 1 or more vaginal suppositories (eg, prostaglandin) with or without cervical dilation (eg, laminaria), including hospital admission and visits, delivery of fetus and secundines; with hysterotomy (failed medical evacuation) |
|  | CPT | 1965 | Anesthesia for incomplete or missed abortion procedures |
|  | CPT | 59812 | Treatment of incomplete abortion, any trimester, completed surgically |
|  | CPT | 59820 | Treatment of missed abortion, completed surgically; first trimester |
|  | CPT | 59821 | Treatment of missed abortion, completed surgically; second trimester |
|  | CPT | 59830 | Treatment of septic abortion, completed surgically |
|  | HCPCS | S2260 | Induced abortion, 17 to 24 weeks |
|  | HCPCS | S0199 | Med abortion inc all ex drug |
|  | HCPCS | S0191 | Misoprostol, oral, 200 mcg |
|  | HCPCS | S0190 | Mifepristone, oral, 200 mg |
|  | ICD9CM | 635 | Legally induced abortion |
|  | ICD9CM | 636 | Illegal abortion |
|  | ICD9CM | 7796 | Termination of pregnancy (fetus) |
|  | ICD9CM | 6310 | Inappropriate change in quantitative human chorionic gonadotropin (hCG) in early pregnancy |
|  | ICD9CM | 6318 | Other abnormal products of conception |
|  | ICD9CM | 632 | Missed abortion |
|  | ICD9CM | 634 | Spontaneous abortion |
|  | ICD9CM | 6370 | Unspecified abortion complicated by genital tract and pelvic infection |
|  | ICD9CM | 6371 | Unspecified abortion complicated by delayed or excessive hemorrhage |
|  | ICD9CM Proc | 6901 | Dilation And Curettage For Termination Of Pregnancy |
|  | ICD9CM Proc | 6951 | Aspiration Curettage Of Uterus For Termination Of Pregnancy |
|  | ICD9CM Proc | 7491 | Hysterotomy To Terminate Pregnancy |
|  | ICD9CM Proc | 750 | Intra-Amniotic Injection For Abortion |
| Ectopic pregnancy | CPT | 59120 | Surgical treatment of ectopic pregnancy; tubal or ovarian, requiring salpingectomy and/or oophorectomy, abdominal or vaginal approach |
|  | CPT | 59121 | Surgical treatment of ectopic pregnancy; tubal or ovarian, without salpingectomy and/or oophorectomy |
|  | CPT | 59130 | Surgical treatment of ectopic pregnancy; abdominal pregnancy |
|  | CPT | 59135 | Surgical treatment of ectopic pregnancy; interstitial, uterine pregnancy requiring total hysterectomy |
|  | CPT | 59136 | Surgical treatment of ectopic pregnancy; interstitial, uterine pregnancy with partial resection of uterus |
|  | CPT | 59140 | Surgical treatment of ectopic pregnancy; cervical, with evacuation |
|  | CPT | 59150 | Laparoscopic treatment of ectopic pregnancy; without salpingectomy and/or oophorectomy |
|  | CPT | 59151 | Laparoscopic treatment of ectopic pregnancy; with salpingectomy and/or oophorectomy |
|  | ICD9CM | 633 | Ectopic pregnancy |
|  | ICD9CM Proc | 743 | Removal Of Extratubal Ectopic Pregnancy |
| Others (Prenatal Visits) | CPT | 59015 | Chorionic villus sampling, any method |
|  | CPT | 76801 | Ultrasound, pregnant uterus, real time with image documentation, fetal and maternal evaluation, first trimester (< 14 weeks 0 days), transabdominal approach; single or first gestation |
|  | CPT | 76802 | Ultrasound, pregnant uterus, real time with image documentation, fetal and maternal evaluation, first trimester (< 14 weeks 0 days), transabdominal approach; each additional gestation (List separately in addition to code for primary procedure) |
|  | CPT | 76945 | Ultrasonic guidance for chorionic villus sampling, imaging supervision and interpretation |
|  | CPT | 84163 | Pregnancy-associated plasma protein-A (PAPP-A) |
|  | CPT | 76813 | Ultrasound, pregnant uterus, real time with image documentation, first trimester fetal nuchal translucency measurement, transabdominal or transvaginal approach; single or first gestation |
|  | CPT | 76814 | Ultrasound, pregnant uterus, real time with image documentation, first trimester fetal nuchal translucency measurement, transabdominal or transvaginal approach; each additional gestation (List separately in addition to code for primary procedure) |
|  | ICD9CM | V22 | Normal pregnancy |
|  | ICD9CM | V23 | Supervision of high-risk pregnancy |
|  | ICD9CM | V7242 | Pregnancy examination or test, positive result |
|  | ICD9CM | V283 | Encounter for routine screening for malformation using ultrasonics |
| Others | CPT | 1960 | Anesthesia for vaginal delivery only |
|  | CPT | 1961 | Anesthesia for cesarean delivery only |
|  | CPT | 1963 | Anesthesia for cesarean hysterectomy without any labor analgesia/anesthesia care |
|  | CPT | 1967 | Neuraxial labor analgesia/anesthesia for planned vaginal delivery (this includes any repeat subarachnoid needle placement and drug injection and/or any necessary replacement of an epidural catheter during labor) |
|  | CPT | 1968 | Anesthesia for cesarean delivery following neuraxial labor analgesia/anesthesia (List separately in addition to code for primary procedure performed) |
|  | CPT | 1969 | Anesthesia for cesarean hysterectomy following neuraxial labor analgesia/anesthesia (List separately in addition to code for primary procedure performed) |
|  | CPT | 58611 | Ligation or transection of fallopian tube(s) when done at the time of cesarean delivery or intra-abdominal surgery (not a separate procedure) (List separately in addition to code for primary procedure) |
|  | CPT | 59300 | Episiotomy or vaginal repair, by other than attending |
|  | CPT | 59400 | Routine obstetric care including antepartum care, vaginal delivery (with or without episiotomy, and/or forceps) and postpartum care |
|  | CPT | 59409 | Vaginal delivery only (with or without episiotomy and/or forceps) |
|  | CPT | 59414 | Delivery of placenta (separate procedure) |
|  | CPT | 59510 | Routine obstetric care including antepartum care, cesarean delivery, and postpartum care |
|  | CPT | 59514 | Cesarean delivery only |
|  | CPT | 59525 | Subtotal or total hysterectomy after cesarean delivery (List separately in addition to code for primary procedure) |
|  | CPT | 59610 | Routine obstetric care including antepartum care, vaginal delivery (with or without episiotomy, and/or forceps) and postpartum care, after previous cesarean delivery |
|  | CPT | 59612 | Vaginal delivery only, after previous cesarean delivery (with or without episiotomy and/or forceps) |
|  | CPT | 59614 | Vaginal delivery only, after previous cesarean delivery (with or without episiotomy and/or forceps); including postpartum care |
|  | CPT | 59618 | Routine obstetric care including antepartum care, cesarean delivery, and postpartum care, following attempted vaginal delivery after previous cesarean delivery |
|  | CPT | 59620 | Cesarean delivery only, following attempted vaginal delivery after previous cesarean delivery |
|  | CPT | 99436 | Attendance at delivery (when requested by delivering physician) and initial stabilization of newborn |
|  | CPT | 99464 | Attendance at delivery (when requested by delivering physician) and initial stabilization of newborn |
|  | CPT | 59622 | Cesarean delivery only, following attempted vaginal delivery after previous cesarean delivery; including postpartum care |
|  | ICD9CM | 650 | Normal delivery |
|  | ICD9CM | 65101 | Twin pregnancy delivered |
|  | ICD9CM | 65111 | Triplet pregnancy delivered |
|  | ICD9CM | 65121 | Quadruplet pregnancy delivered |
|  | ICD9CM | 65151 | Quadruplet pregnancy with fetal loss and retention of one or more fetus(es) delivered with or without antepartum condition |
|  | ICD9CM | 65901 | Failed mechanical induction of labor, delivered, with or without mention of antepartum condition |
|  | ICD9CM | 66051 | Locked twins with delivery |
|  | ICD9CM | 66061 | Unspecified failed trial of labor with delivery |
|  | ICD9CM | 66071 | Unspecified failed forceps or vacuum extractor with delivery |
|  | ICD9CM | 66081 | Other causes of obstructed labor with delivery |
|  | ICD9CM | 66091 | Unspecified obstructed labor with delivery |
|  | ICD9CM | 66401 | First-degree perineal laceration with delivery |
|  | ICD9CM | 66411 | Second-degree perineal laceration with delivery |
|  | ICD9CM | 66421 | Third-degree perineal laceration with delivery |
|  | ICD9CM | 66431 | Fourth-degree perineal laceration with delivery |
|  | ICD9CM | 66441 | Unspecified perineal laceration with delivery |
|  | ICD9CM | 66451 | Vulvar and perineal hematoma with delivery |
|  | ICD9CM | 66481 | Other specified trauma to perineum and vulva with delivery |
|  | ICD9CM | 66491 | Unspecified trauma to perineum and vulva with delivery |
|  | ICD9CM | 66501 | Rupture of uterus before onset of labor with delivery |
|  | ICD9CM | 66511 | Rupture of uterus during labor with delivery |
|  | ICD9CM | 66531 | Obstetrical laceration of cervix with delivery |
|  | ICD9CM | 66541 | High vaginal laceration during and after labor with delivery |
|  | ICD9CM | 66581 | Other specified obstetrical trauma with delivery |
|  | ICD9CM | 66591 | Unspecified obstetrical trauma with delivery |
|  | ICD9CM | 66951 | Forceps or vacuum extractor delivery without indication delivered with or without antepartum condition |
|  | ICD9CM | 66961 | Breech extraction without indication delivered with or without antepartum condition |
|  | ICD9CM | 66971 | Cesarean delivery without indication delivered with or without antepartum condition |
|  | ICD9CM Proc | 72 | Forceps, Vacuum, And Breech Delivery |
|  | ICD9CM Proc | 720 | Low Forceps Operation |
|  | ICD9CM Proc | 721 | Low Forceps Operation With Episiotomy |
|  | ICD9CM Proc | 722 | Mid Forceps Operation |
|  | ICD9CM Proc | 7221 | Mid Forceps Operation With Episiotomy |
|  | ICD9CM Proc | 7229 | Other Mid Forceps Operation |
|  | ICD9CM Proc | 723 | High Forceps Operation |
|  | ICD9CM Proc | 7231 | High Forceps Operation With Episiotomy |
|  | ICD9CM Proc | 7239 | Other High Forceps Operation |
|  | ICD9CM Proc | 724 | Forceps Rotation Of Fetal Head |
|  | ICD9CM Proc | 725 | Breech Extraction |
|  | ICD9CM Proc | 7251 | Partial Breech Extraction With Forceps To Aftercoming Head |
|  | ICD9CM Proc | 7252 | Other Partial Breech Extraction |
|  | ICD9CM Proc | 7253 | Total Breech Extraction With Forceps To Aftercoming Head |
|  | ICD9CM Proc | 7254 | Other Total Breech Extraction |
|  | ICD9CM Proc | 726 | Forceps Application To Aftercoming Head |
|  | ICD9CM Proc | 727 | Vacuum Extraction |
|  | ICD9CM Proc | 7271 | Vacuum Extraction With Episiotomy |
|  | ICD9CM Proc | 7279 | Other Vacuum Extraction |
|  | ICD9CM Proc | 728 | Other Specified Instrumental Delivery |
|  | ICD9CM Proc | 729 | Unspecified Instrumental Delivery |
|  | ICD9CM Proc | 73 | Other Procedures Inducing Or Assisting Delivery |
|  | ICD9CM Proc | 730 | Artificial Rupture Of Membranes |
|  | ICD9CM Proc | 7301 | Induction Of Labor By Artificial Rupture Of Membranes |
|  | ICD9CM Proc | 7309 | Other Artificial Rupture Of Membranes |
|  | ICD9CM Proc | 731 | Other Surgical Induction Of Labor |
|  | ICD9CM Proc | 732 | Internal And Combined Version And Extraction |
|  | ICD9CM Proc | 7321 | Internal And Combined Version Without Extraction |
|  | ICD9CM Proc | 7322 | Internal And Combined Version With Extraction |
|  | ICD9CM Proc | 733 | Failed Forceps |
|  | ICD9CM Proc | 734 | Medical Induction Of Labor |
|  | ICD9CM Proc | 735 | Manually Assisted Delivery |
|  | ICD9CM Proc | 7351 | Manual Rotation Of Fetal Head |
|  | ICD9CM Proc | 7359 | Other Manually Assisted Delivery |
|  | ICD9CM Proc | 736 | Episiotomy |
|  | ICD9CM Proc | 738 | Operations On Fetus To Facilitate Delivery |
|  | ICD9CM Proc | 739 | Other Operations Assisting Delivery |
|  | ICD9CM Proc | 7391 | External Version |
|  | ICD9CM Proc | 7392 | Replacement Of Prolapsed Umbilical Cord |
|  | ICD9CM Proc | 7393 | Incision Of Cervix To Assist Delivery |
|  | ICD9CM Proc | 7394 | Pubiotomy To Assist Delivery |
|  | ICD9CM Proc | 7399 | Other Procedures Inducing Or Assisting Delivery |
|  | ICD9CM Proc | 74 | Cesarean Section And Removal Of Fetus |
|  | ICD9CM Proc | 740 | Classical Cesarean Section |
|  | ICD9CM Proc | 741 | Low Cervical Cesarean Section |
|  | ICD9CM Proc | 742 | Extraperitoneal Cesarean Section |
|  | ICD9CM Proc | 744 | Cesarean Section Of Other Specified Type |
|  | ICD9CM Proc | 749 | Cesarean Section Of Unspecified Type |
|  | ICD9CM Proc | 7499 | Other Cesarean Section Of Unspecified Type |
|  | ICD9CM Proc | 754 | Manual Removal Of Retained Placenta |
|  | ICD9CM Proc | 755 | Repair Of Current Obstetric Laceration Of Uterus |
|  | ICD9CM Proc | 7550 | Repair Of Current Obstetric Laceration Of Uterus Not Otherwise Specified |
|  | ICD9CM Proc | 7551 | Repair Of Current Obstetric Laceration Of Cervix |
|  | ICD9CM Proc | 7552 | Repair Of Current Obstetric Laceration Of Corpus Uteri |
|  | ICD9CM Proc | 756 | Repair Of Other Current Obstetric Laceration |
|  | ICD9CM Proc | 7561 | Repair Of Current Obstetric Laceration Of Bladder And Urethra |
|  | ICD9CM Proc | 7562 | Repair Of Current Obstetric Laceration Of Rectum And Sphincter Ani |
|  | ICD9CM Proc | 7569 | Repair Of Other Current Obstetric Laceration |
|  | ICD9CM Proc | 757 | Manual Exploration Of Uterine Cavity, Postpartum |

ICD-9-CM: international classification of disease, ninth revision, clinical modification, diagnosis; CPT: Current Procedural Terminology; ICD9CM Proc: international classification of disease, ninth revision, clinical modification, procedures; HCPCS: Healthcare Common Procedure Coding System
