# Supplementary material for: Comparing pregnancy and pregnancy outcome rates between adolescents with and without pre-existing mental disorders
Source: PLoS One. 2024 Mar 14;19(3):e0296425. doi: 10.1371/journal.pone.0296425 (PMC10939254; doi:10.1371/journal.pone.0296425)
Supplement: S2 Table — The list of ICD-9-CM codes categorized by 15 mental disorders based on the Clinical Classifications Software (CCS) developed by the Agency for Healthcare Research and Quality (AHRQ). (DOCX) [file pone.0296425.s002.docx]

| S2 Table. The fifteen mental disorder categories* | | |  | |
| --- | --- | --- | --- | --- |
| Mental disorder | ICD-9-CM code | CCS code | |  |
| Adjustment Disorders | 309.1, 309.0, 309.22, 309.23, 309.24, 309.28, 309.29, 309.3, 309.4, 309,82, 309.83, 309.89, 309.9 | 650 | |  |
| Alcohol-Related Disorders | 291.0, 291.1, 291.2, 291.3, 2914, 291.5, 291.8, 291.81, 291.82, 291.89, 291.9, 303.00, 303.01, 303.02, 30303, 303.90, 303.91, 303.92, 303.93, 305.00, 305.01, 305.02, 305.03, 357.5, 425.5, 535.3, 535.30, 53531, 5710, 5711, 571.2, 571.3, 760.71, 980.0 | 660 | |  |
| Anxiety Disorders | 293.84, 300.00, 300.01, 300.02, 300.09, 300.10, 300.20, 300.21, 300.22, 300.23, 300.29, 300.3,  300.5, 300.89, 300.9, 308.0, 308.1, 308.2, 308.3, 308.4, 308.9, 309.81, 313.0, 313.1, 313.21, 313.22, 313.3, 313.82, 313.83 | 651 | |  |
| Attention-Deficit/Conduct/Disruptive Behavior Disorders | 312.00, 312.01, 312.02, 312.03, 312.10, 312.11, 312.12, 312.13, 312.20, 312.21, 312.22, 312.23, 312.4, 312.8, 312.81, 312.82, 312.89, 312.9, 313.81, 314.00, 314.01, 314.1, 314.2, 314.8, 314.9 | 652 | |  |
| Delirium/Dementia/Amnestic/Other Cognitive Disorders | 290.0, 290.10, 290.11, 290.12, 290.13, 290.20, 290.21, 290.3, 290.40, 290.41, 290.42, 290.43, 290.8, 290.9, 293.0, 293.1, 294.0, 294.1, 294.10, 294.11, 294.20, 294.21, 294.8, 294.9, 310.0, 310.2, 310.8, 310.81, 31089, 310.9, 331.0, 331.1, 331.11, 331.19, 331.2, 331.82, 797 | 653 | |  |
| Developmental Disorders | 307.0, 307.9, 315.31, 315.34, 315.35, 315.39, V40.1, 315.01, 315.02, 315.09, 315.32, 315.5, 315.8, 317, 3180, 318.1, 318.2, 319, 315.00, 315.1, 315.2, 315.9, V40.0, 315.4 | 654 | |  |
| Disorders Usually Diagnosed In Infancy, Childhood, or Adolescence | 307.6, 307.7, 307.3, 309.21, 313.23, 313.89, 313.9, 299.00, 299.01, 299.10, 299.11, 299.80, 299.81, 299.90, 299.91, 307.20, 307.21, 307.22, 307.23 | 655 | |  |
| Impulse Control Disorders Not Elsewhere Classified | 312.30, 312.31, 312.32, 312.33, 312.34, 312.35, 312.39 | 656 | |  |
| Miscellaneous Mental Disorders : Dissociative disorders, Eating disorders, Factitious disorders, Psychogenic disorders, Sexual and gender identity disorders, Sleep disorders, Somatoform disorders, Mental disorders due to general medical conditions not elsewhere classified | 300.12, 300.13, 300.14, 300.15, 300.6, 307.1, 307.50, 307.51, 307.52, 307.53, 307.54, 307.59, 300.16, 300.19, 306.0, 306.1, 306.2, 306.3, 306.4, 306.50, 306.52, 306.53, 306.59, 306.6, 306.7, 306.8, 306.9, 302.1, 302.2, 302.3, 302.4, 302.50, 302.51, 302.52, 302.53, 302.6, 302.70, 302.71, 302.72, 302.73, 302.74, 302.75, 302.76, 302.79, 302.81, 302.82, 302.83, 302.84, 302.85, 302.89, 302.9, 306.51, 307.40, 307.41, 307.42, 307.43, 307.44, 307.45, 307.46, 307.47, 307.48, 307.49, 300.11, 300.7, 300.81, 300.82, 307.80, 307.81, 307.89, 293.89, 293.9, 310.1, 316, 648.40, 648.41, 648.42, 648.43, 648.44, V40.2, V40.3, V40.31, V40.39, V40.9, V67.3 | 670 | |  |
| Mood Disorders – Depressive Disorders | 293.83, 296.20, 296.21, 296.22, 296.23, 296.24, 296.25, 296.26, 296.30, 296.31, 296.32, 296.33,  296.34, 296.35, 296.36, 300.4, 311 | 657.2 | |  |
| Mood Disorders – Bipolar Disorders | 296.00, 296.01, 296.02, 296.03, 296.04, 296.05, 296.06, 296.10, 296.11, 296.12, 296.13, 296.14,  296.15, 296.16, 296.40, 296.41, 296.42, 296.43, 296.44, 296.45, 296.46, 296.50, 296.51, 296.52,  296.53, 296.54, 296.55, 296.56, 296.60, 296.61, 296.62, 296.63, 296.64, 296.65, 296.66, 296.7,  296.80, 296.81, 296.82, 296.89, 296.90, 296.99 | 657.1 | |  |
| Personality Disorders | 301.0, 301.10, 301.11, 301.12, 301.13, 301.20, 301.21, 301.22, 301.3, 301.4, 301.50, 301.51,  301.59, 301.6, 301.7, 301.81, 301.82, 301.83, 301.84, 301.89, 301.9 | 658 | |  |
| Schizophrenia and Other Psychotic Disorders | 293.81, 293.82, 295.00, 295.01, 295.02, 295.03, 295.04, 295.05, 295.10, 295.11, 295.12, 295.13,  295.14, 295.15, 295.20, 295.21, 295.22, 295.23, 295.24, 295.25, 295.30, 295.31, 295.32, 295.33,  295.34, 295.35, 295.40, 295.41, 295.42, 295.43, 295.44, 295.45, 295.50, 295.51, 295.52, 295.53,  295.54, 295.55, 295.60, 295.61, 295.62, 295.63, 295.64, 295.65, 295.70, 295.71, 295.72, 295.73,  295.74, 295.75, 295.80, 295.81, 295.82, 295.83, 295.84, 295.85, 295.90, 295.91, 295.92, 295.93,  295.94, 295.95, 297.0, 297.1, 297.2, 297.3, 297.8, 297.9, 298.0, 298.1, 298.2, 298.3, 298.4, 298.8, 298.9 | 659 | |  |
| Substance-Related Disorders | 292.0, 292.11, 292.12, 292.2, 292.81, 292.82, 292.83, 292.84, 292.85, 292.89, 292.9, 304.00, 304.01, 304.02, 304.03, 304.10, 304.11, 304.12, 304.13, 304.20, 304.21, 304.22, 304.23, 304.30, 304.31, 304.32, 304.33, 304.40, 304.41, 304.42, 304.43, 304.50, 304.51, 304.52, 304.53, 304.60, 304.61, 304.62, 304.63, 304.70, 304.71, 304.72, 304.73, 304.80, 304.81, 304.82, 304.83, 304.90, 304.91, 304.92, 304.93, 305.20, 305.21, 305.22, 305.23, 305.30, 305.31, 305.32, 305.33, 305.40, 305.41, 305.42, 305.43, 305.50, 305.51, 305.52, 305.53, 305.60, 305.61, 305.62, 305.63, 305.70, 305.71, 305.72, 305.73, 305.80, 305.81, 305.82, 305.83, 305.90, 305.91, 305.92, 305.93, 648.30, 648.31, 648.32, 648.33, 648.34, 655.50, 655.51, 655.53, 760.72, 760.73, 760.75, 779.5, 965.00, 965.01, 965.02, 965.09, V65.42 | 661 | |  |
| Suicide and Intentional Self-Inflicted Injury | E950.0, E950.1, E950.2, E950.3, E950.4, E950.5, E950.6, E950.7, E950.8, E950.9, E951.0, E951.1, E951.8, E952.0, E952.1, E952.8, E952.9, E953.0, E953.1, E953.8, E953.9, E954, E955.0, E955.1, E955.2, E955.3, E955.4, E955.5, E955.6, E955.7, E955.9, E956, E957.0, E957.1, E957.2, E957.9, E958.0, E958.1, E958.2, E958.3, E958.4, E958.5, E958.6, E958.7, E958.8, E958.9, E959, V62.84 | 662 | |  |

* based on the Clinical Classifications Software (CCS) developed by the Agency for Healthcare Research and Quality (AHRQ)

CCS: Clinical Classifications Software
