# Supplementary figures and images for: Comparing pregnancy and pregnancy outcome rates between adolescents with and without pre-existing mental disorders
Source: PLoS One. 2024 Mar 14;19(3):e0296425. doi: 10.1371/journal.pone.0296425 (PMC10939254; doi:10.1371/journal.pone.0296425)

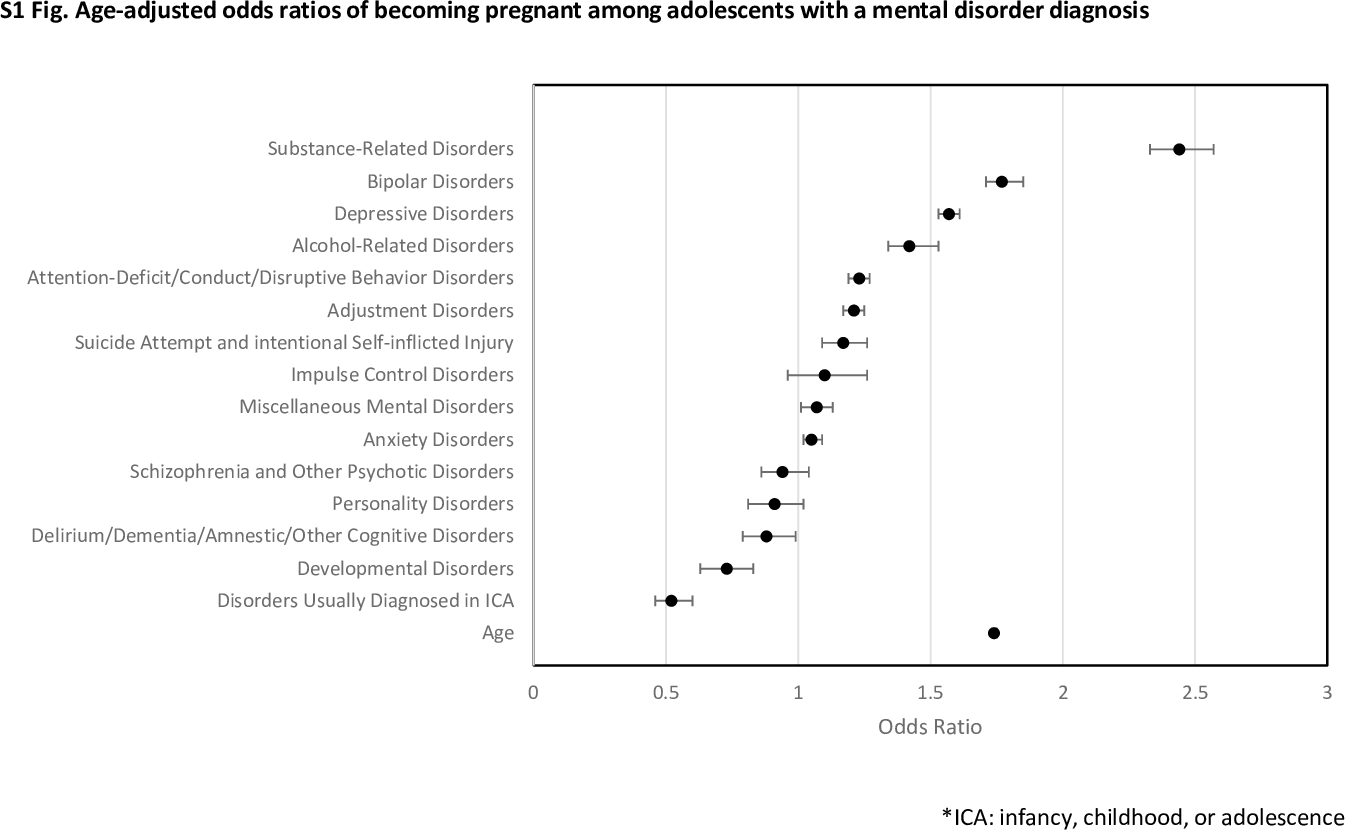

Supplement: S1 Fig — (TIF) [file pone.0296425.s003.tif]

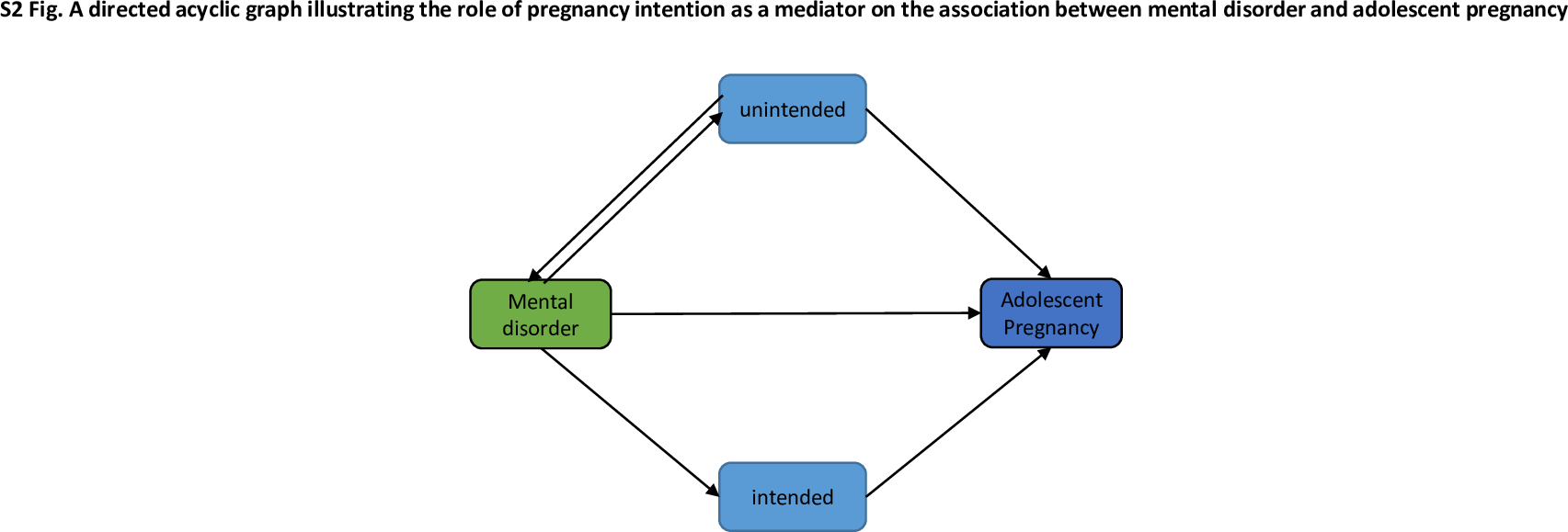

Supplement: S2 Fig — (TIF) [file pone.0296425.s004.tif]
