# Supplementary material for: Comparing pregnancy and pregnancy outcome rates between adolescents with and without pre-existing mental disorders
Source: PLoS One. 2024 Mar 14;19(3):e0296425. doi: 10.1371/journal.pone.0296425 (PMC10939254; doi:10.1371/journal.pone.0296425)
Supplement: Attachment — Submitted filename: Response to Reviewers.docx [file pone.0296425.s007.docx]

Academic Editor’s comments: Please review your reference list to ensure that it is complete and correct. If you have cited papers that have been retracted, please include the rationale for doing so in the manuscript text, or remove these references and replace them with relevant current references. Any changes to the reference list should be mentioned in the rebuttal letter that accompanies your revised manuscript. If you need to cite a retracted article, indicate the article’s retracted status in the References list and also include a citation and full reference for the retraction notice.

We reviewed the reference list to ensure it is complete and correct. Also, there were no references that have been retracted.

References #11, #14, #20, #21, and #32 were re-formatted to meet the submission guideline. (See below). As for the reference #21, we have updated the URL as the address has been changed since we checked it last time.

11. Sekharan VS, Kim TH, Oulman E, Tamim H. Prevalence and characteristics of intended adolescent pregnancy: an analysis of the Canadian maternity experiences survey. Reprod Health. 2015;12:101.

14.Merative™ MarketScan Research Databases. [Cited 2023 Sep 26]. Available from: <https://www.merative.com/real-world-evidence>.

20.Files H. Clinical Classifications Software (CCS) for ICD-9-CM: Agency for healthcare research and quality. [Cited 2023 Sep 26]. Available from: <https://www.hcup-us.ahrq.gov/toolssoftware/ccs/ccs.jsp>.

21.Centers for Disease Control and Prevention. Annual Estimates of the resident population by single year of age and sex for the United States. U.S. Department of Health & Human Services. [Cited 2023 Sep 26]. Available from: <https://catalog.data.gov/dataset/us-census-annual-estimates-of-the-resident-population-for-selected-age-groups-by-sex-for-t>.

32.Kost K M-ZI, Arpaia A. Pregnancies, births and abortions among adolescents and young women in the United States: Guttmacher Institute; 2013. [Cited 2023 Sep 26]. Available from: <https://www.guttmacher.org/sites/default/files/report_pdf/us-adolescent-pregnancy-trends-2013.pdf>.
