# Supplementary material for: Comparing pregnancy and pregnancy outcome rates between adolescents with and without pre-existing mental disorders
Source: PLoS One. 2024 Mar 14;19(3):e0296425. doi: 10.1371/journal.pone.0296425 (PMC10939254; doi:10.1371/journal.pone.0296425)
Supplement: Attachment — Submitted filename: Response to Reviewers.docx [file pone.0296425.s008.docx]

Reviewer #2 Comment: In response to earlier comments, the authors included a paragraph in the introduction (line 57 - 66) that I feel is unnecessary given that the data used do not provide details on whether pregnancies were intended or unintended. The authors already clearly articulate the lack of data on pregnancy as a limitation. What they have written in the discussion is adequate.

The lines 57-66 are now removed and along with the removal, the ref 10 is relocated to ref 30 and ref 11 is deleted. In addition, we updated the reference numbering as follow;

-	References 12-31 are renumbered to ref 10-29

-	References 32&33 are now ref 31&32
